# Associations of micronutrient dietary patterns with sarcopenia among US adults: a population-based study

**DOI:** 10.3389/fnut.2024.1301831

**Published:** 2024-02-12

**Authors:** Yining Liu, Xiangliang Liu, Linnan Duan, Yixin Zhao, Yuwei He, Wei Li, Jiuwei Cui

**Affiliations:** ^1^Cancer Center, The First Hospital of Jilin University, Changchun, China; ^2^Department of Neurosurgery, The First Hospital of Jilin University, Changchun, China

**Keywords:** micronutrient, sarcopenia, skeletal muscle mass, principle component analysis, cross-sectional survey

## Abstract

**Background:**

Current epidemiological evidence points to an association between micronutrient (MN) intake and sarcopenia, but studies have focused on single MN, and no combined effects on MNs have been reported. The aim of this study was to investigate the relationship between different MN intake patterns and sarcopenia and skeletal muscle mass.

**Methods:**

We performed a population-based cross-sectional study, with a total of 5,256 U.S. adults aged 20–59 years, and we collected total daily MN intake and appendicular skeletal muscle mass measured by Dual-Energy X-ray Absorptiometry (DXA). Principal component analysis (PCA) was used to obtain nutrient patterns and principal component scores based on the intake of 14 MNs, and logistic regression analysis was used to assess the effects of single MN and MN intake patterns on sarcopenia and muscle mass.

**Results:**

We defined three MN intake patterns by PCA: (1) adherence to VitB-mineral, high intake of vitamin B and minerals; (2) adherence to VitAD-Ca-VB12, high intake of vitamin A, vitamin D, calcium and vitamin B12; and (3) adherence to Antioxidant Vit, high intake of antioxidant vitamins A, C, E, and K. These three nutrient patterns explained 73.26% of the variance of the population. A negative association was observed between most single MN intakes and sarcopenia, and after adjusting for confounders, adherence to the highest tertile of the three nutrient patterns was associated with a lower risk of sarcopenia and relatively higher skeletal muscle mass compared to the lowest adherence. In subgroup analysis, MN intake patterns were significantly correlated with sarcopenia in middle-aged females.

**Conclusion:**

Nutritional patterns based on MN intake were significantly related to sarcopenia, indicating that MNs interact with each other while exerting their individual functions, and that MN dietary patterns may provide promising strategies for preventing the loss of muscle mass, with further prospective studies warranted in the future.

## 1 Introduction

Sarcopenia refers to the progressive decline of skeletal muscle mass and strength associated with aging (1). As a major contributor to physical frailty and disability in later life, sarcopenia has become a critical public health challenge with the rapid growth of aging population worldwide (2, 3). The etiology and pathogenesis of sarcopenia are multi-factorial, involving skeletal muscle changes, neurological deterioration, endocrine disorder, chronic inflammation, oxidative stress, malnutrition, and reduced physical activity (4). Nutritional intervention is one of the cornerstones in the management of sarcopenia, whereas greater attention is focused on protein and energy metabolism as well as the role of essential amino acids and their metabolites. In recent years, inadequate dietary intake and status of micronutrients (MNs) including vitamins and minerals have emerged as potentially modifiable risks for sarcopenia (5), which implies that MNs could be a promising target for sarcopenia intervention.

MNs deficiency is a nutritional and health problem of the world, and has been associated with a wide range of diseases and frailty (6). Supplementation with MNs is an important part of enteral nutrition (EN) and parenteral nutrition (PN). MNs such as vitamin D (VD), antioxidants, B vitamins and minerals play indispensable roles in maintaining muscle health via regulating mitochondrial biogenesis, redox homeostasis, protein metabolism and neuromuscular function (7–10). For example, several population-based studies have found an association of reduced levels of B vitamins and VD and myasthenia and muscle mass loss (11–14). Observational studies have associated insufficient blood levels or dietary consumption of antioxidants (15) and minerals (16, 17) with accelerated loss of muscle mass and strength. However, intervention trials testing single MN supplementation show inconsistent results (18, 19), and available evidence for individual MN remains limited and mixed.

Dietary pattern analysis provides a comprehensive approach to examine cumulative and interactive effects of overall food and nutrient intake (20). Recent research applying this methodology has revealed healthful dietary patterns abundant in fruits, vegetables, fish and whole grains may help preserve muscle integrity in older adults (21). Nonetheless, studies delineating the relationship between whole-diet MN patterns and sarcopenia prevalence are still scarce. Furthermore, the potential impact of MN intake on muscle aging process across life course is much less studied compared to old age (22). Sarcopenia is increasingly recognized to develop progressively from midlife, concurrent with decline and imbalance of multiple physiological systems (23, 24). Ensuring adequate MN status starting from early stages may help mitigate sarcopenic risk (25). Using National Health and Nutrition Examination Survey (NHANES) 2011–2014, this present cross-sectional study for the first time identified three major dietary MN patterns and examined their associations with sarcopenia prevalence and muscle mass in US adults aged 20–59 years. Elucidating the link between MN patterns and muscle aging in younger and middle-aged populations would provide significant insights into possible timely nutritional strategies against sarcopenia across adulthood.

## 2 Methods

### 2.1 Study design and participants

**Figure 1:**
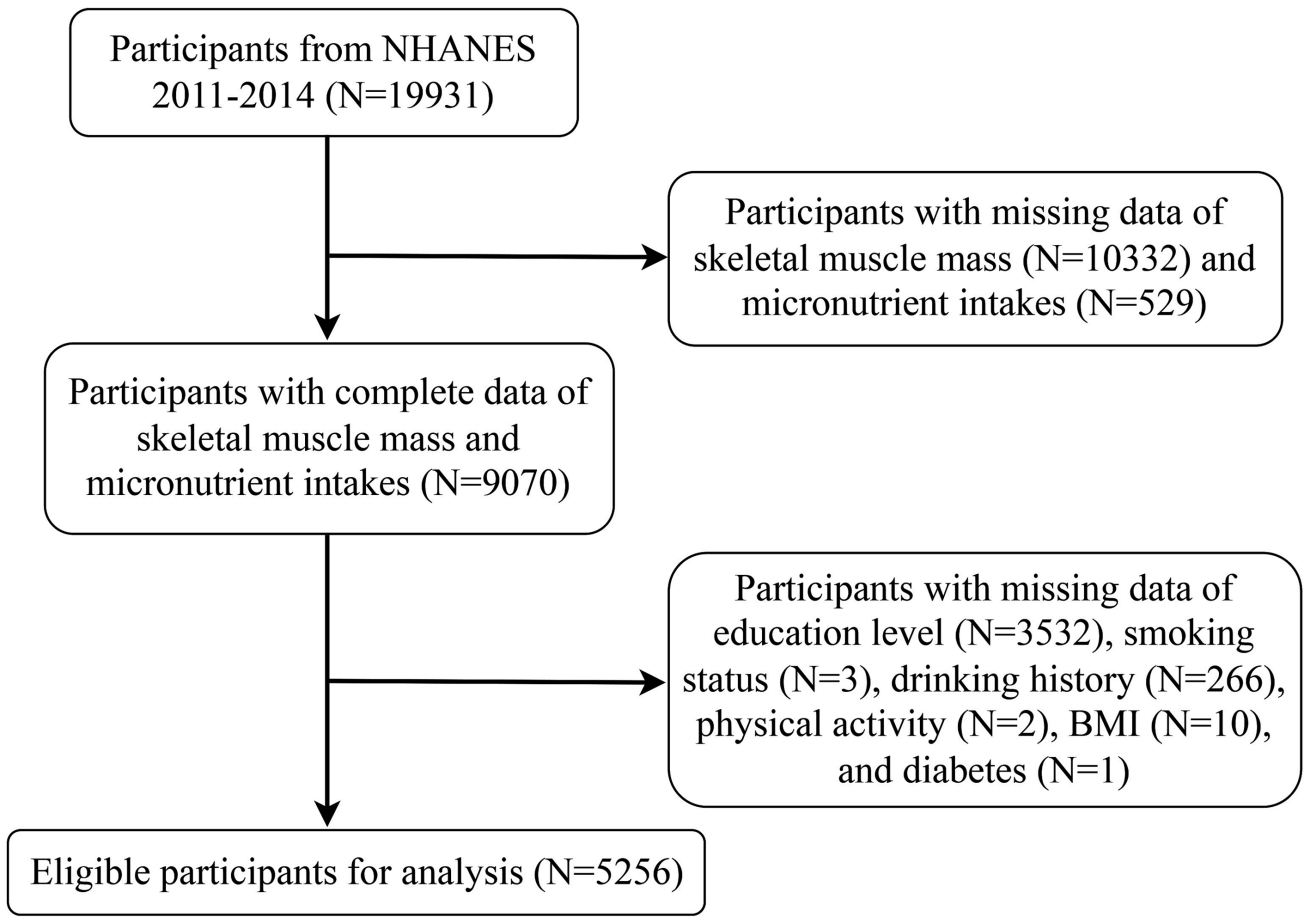
Flow diagram for the selection of participants in NHANES 2011–2014.

The cross-sectional study collected NHANES data representing the national non-institutionalized U.S. population in two cycles from 2011 to 2014, which were obtained through household interviews, Mobile Examination Center (MEC), and laboratory tests.^1^ A total of 19,931 participants were sampled, with the following inclusion criteria: (1) adults aged≥20 years and < 60 years; (2) with complete dietary intake and Dual-Energy X-ray Absorptiometry (DXA) whole-body scans; and (3) with complete baseline characteristics and questionnaires. The flow diagram of selection and exclusion of participants was shown in Figure 1, with a total of 5,256 participants enrolled in the study eventually. The NHANES protocol was approved by the Research Ethics Review Board of the National Center for Health Statistics (NCHS), and all participants signed informed consent forms.

### 2.2 Nutrient intake measurement

During the survey, a 24-h dietary recall was conducted by trained dietary interviewers to obtain Total Nutrient Intakes for the first 24 h collected by the MEC. We collected intakes of vitamins including Vitamin A, niacin (Vitamin B3, VB3), Vitamin B6 (VB6), folate (Vitamin B9, VB9), Vitamin B12 (VB12), Vitamin C, VD, Vitamin E, Vitamin K, and minerals including calcium (Ca), phosphorus (P), iron (Fe), zinc (Zn), and selenium (Se) of each participant. In addition, we obtained the intake of four macronutrients (energy, protein, carbohydrates, and total fat) from all subjects. The dietary data were collected and estimated jointly by the U.S. Department of Agriculture (USDA) and the U.S. Department of Health and Human Services (DHHS).

### 2.3 Assessment of sarcopenia

Appendicular skeletal muscle mass (ASM) were measured using DXA whole-body scans, which were obtained on a Hologic Discovery Model A densitometer (Hologic, Inc., Bedford, Massachusetts) using software version Apex 3.2. More detailed information of the DXA examination protocol is documented on the NHANES website.^2^ Appendicular lean tissue mass (ALM) was obtained by summing the lean tissue mass of the four limbs, and the ratio of ALM (kg) to body mass index (BMI, kg/m^2^) was used to define sarcopenia (<0.789 in males and <0.512 in females) according to the diagnostic criteria of the Foundation for the National Institutes of Health (FNIH) (26), which was used in several studies (27, 28).

### 2.4 Covariates

Data on sociodemographic and individual behavioral characteristics, including age, sex, race, BMI, education level, smoking status, drinking history, recreational physical activity, and comorbidities (hypertension, diabetes, cardiovascular disease, and cancer) were collected through household interviews. The populations were categorized into 4 racial groups (non-Hispanic white, non-Hispanic black, Mexican American, and others) and 3 educational groups (less than high school, high school, and above high school). BMI was calculated by dividing weight (kg) by square of height (m^2^) and people were divided into underweight (<18.5 kg/m^2^), normal weight (18.5–24.9 kg/m^2^), overweight (25–29.9 kg/m^2^) and obese (≥30 kg/m^2^). Smoking status was classified into three categories: never (smoking less than 100 cigarettes in life), ever (smoking more than 100 cigarettes in life, currently do not smoke at all), and now (smoking more than 100 cigarettes in life, currently smoke). People were categorized into drinkers and non-drinkers based on whether they had more than 12 drinks in 1 year. Recreational physical activity was divided into sedentary (MET = 0), insufficient (0 < MET≤500), moderate (500 < MET≤1,000), and high (MET>1,000) by metabolic equivalent (MET)-minutes per week.

Diagnostic criteria for hypertension: (1) Have you been told by a doctor or health professional that you have hypertension? (2) Ever used antihypertensive drugs; (3) Systolic blood pressure ≥ 140 mmHg and diastolic blood pressure ≥ 90 mmHg in three blood pressure measurements.

Diagnostic criteria for diabetes: (1) Have you been told by a doctor or health professional that you have diabetes? (2) Glycosylated hemoglobin (HbA1c) ≥6.5 mmol/L; (3) Fasting blood glucose (GHLU) ≥ 7.0 mmol/L; (4) Ever used anti-diabetic drugs.

### 2.5 Statistical analysis

**Figure 2:**
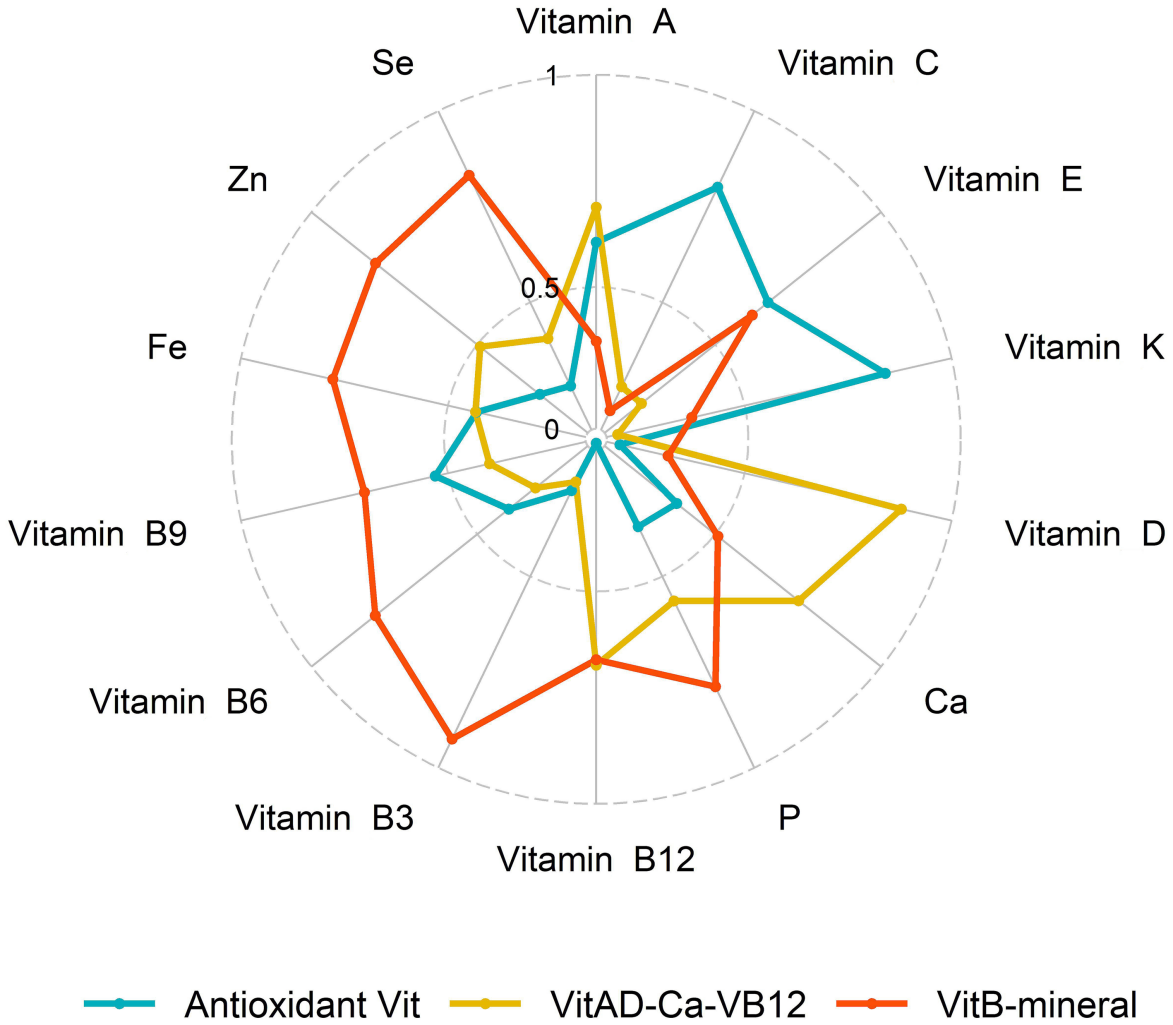
Factor loadings of micronutrients corresponding to micronutrient patterns.

We used principal component analysis (PCA) to assess MN intake patterns of participants over 2 cycles, vitamins and minerals were log-transformed and normalized sequentially for factor analysis. Sample adequacy was tested using the Kaiser-Meyer-Olkin (KMO) test, and varimax rotation was performed using the maximum variance method to obtain nutrient-based factor loadings. A scree plot was used to identify major patterns (eigenvalues >1), and the top 3 principal components with relatively high variance explained were selected as the main MN patterns (Figure 2). Nutrients with factor loadings ≥0.4 were considered to be major contributors. Then we calculated the principal component scores for each pattern, and each principal component score was categorized into tertiles for subsequent statistical analyses, with the highest tertile of participants considered most adherent to this nutrient intake pattern.

MN and macronutrient intakes were all log-transformed to ensure normal distribution, and one-way analysis of variance (ANOVA) and chi-square tests were used to characterize differences in baseline characteristics. MN and macronutrient intakes were expressed as mean (standard error, SE), and demographic characteristics were expressed as median (interquartile, IQR) or number (percentage). Restricted cubic spline (RCS) was used to examine the non-linear relationship between single MN and sarcopenia. Multivariate logistic regression was used to analyze the relationship between single MN and nutrient intake patterns and sarcopenia, and linear regression was used to assess the relationship between nutrient patterns and BMI-corrected ASM. Here we controlled for confounders. Model 1: adjusted for sex, age, race, and BMI. Model 2: adjusted for model 1 and education level, physical activity, smoking status, and drinking history. Model 3: adjusted for model 2 and hypertension, diabetes, cardiovasular disease, and cancer. In addition, we conducted subgroup analyses for age and sex, separately. *p*-values were considered significant at <0.05. All analyses were performed using SPSS 25.0 and R Studio (Version 4.3.2).

## 3 Results

### 3.1 Baseline characteristics of all participants

Five thousand two hundred and fifty-six participants were enrolled in the study, ranging in age from 20 to 59 years, with a predominance of non-Hispanic whites and approximately the same proportion of males and females. The majority (82.67%) had a high school or higher education, and more than half of the populations (67.87%) were overweight or obese. A total of 398 (7.57%) had sarcopenia, and age, race, education, BMI, smoking status, drinking history, physical activity, and hypertension, diabetes, and cardiovascular disease differed significantly between sarcopenic and non-sarcopenic populations, as detailed in Table 1.

**Table 1:** Baseline characteristics of the total population and grouped by sarcopenia.

Characteristics, *N* (%)	Overall (*N* = 5,256)	Sarcopenia	Non-sarcopenia	*p* value
Age (years)				<0.001
≥40	2,530 (48.14%)	245 (61.6%)	2,285 (47.0%)	
<40	2,726 (51.86%)	153 (38.4%)	2,573 (53.0%)	
Sex, male	2,698 (51.33%)	199 (50.0%)	2,499 (51.4%)	0.580
Race				<0.001
Mexican American	665 (12.65%)	113 (28.4%)	552 (11.4%)	
Non-Hispanic White	2097 (39.90%)	138 (34.7%)	1959 (40.3%)	
Non-Hispanic Black	1,151 (21.90%)	30 (7.5%)	1,121 (23.1%)	
Others	1,343 (25.55%)	117 (29.4%)	1,226 (25.2%)	
Education level				<0.001
Below high school	911 (17.33%)	103 (25.9%)	808 (16.6%)	
High school	1,132 (21.54%)	105 (26.4%)	1,027 (21.1%)	
Above high school	3,213 (61.13%)	190 (47.7%)	3,023 (62.2%)	
BMI				<0.001
<18.5 kg/m^2^	95 (1.81%)	3 (0.8%)	92 (1.9%)	
18.5–24.9 kg/m^2^	1,594 (30.33%)	31 (7.8%)	1,563 (32.2%)	
25–29.9 kg/m^2^	1,696 (32.27%)	91 (22.9%)	1,605 (33.0%)	
≥30 kg/m^2^	1871 (35.60%)	273 (68.6%)	1,598 (32.9%)	
Smoking status				0.001
Never	3,131 (59.57%)	249 (62.6%)	2,882 (59.3%)	
Ever	876 (16.67%)	82 (20.6%)	794 (16.3%)	
Current	1,249 (23.76%)	67 (16.8%)	1,182 (24.3%)	
Drinking history (yes)	4,048 (77.02%)	266 (66.8%)	3,782 (77.9%)	<0.001
Recreational physical activity				<0.001
Sedentary	2,888 (54.95%)	248 (62.3%)	2,640 (54.3%)	
Insufficient	1,175 (22.36%)	55 (13.8%)	1,120 (23.1%)	
Moderate	652 (12.40%)	54 (13.6%)	598 (12.3%)	
High	541 (10.29%)	41 (10.3%)	500 (10.3%)	
Hypertension (yes)	1,312 (24.96%)	144 (36.2%)	1,168 (24.0%)	<0.001
Diabetes (yes)	674 (12.82%)	107 (26.9%)	567 (11.7%)	<0.001
Cardiovasular disease (yes)	96 (1.83%)	15 (3.8%)	81 (1.7%)	0.003
Cancer (yes)	203 (3.86%)	19 (4.8%)	184 (3.8%)	0.326

Values were presented as number (%). BMI, body mass index.

### 3.2 MN-intake patterns assessment

We identified three major MN intake patterns using PCA with a KMO test of 0.889. Table 2 showed the matrix of all MN compositions as well as their respective factor loadings. The VitB-mineral pattern was characterized by high intake of VB and minerals, and the VitAD-Ca-VB12 pattern was high in VA, VD, Ca and VB12. The Antioxidant Vit pattern showed high positive factor loadings of VA, VC, VE and VK possessing antioxidant properties. These three recognizable patterns explained 73.26% of the variance. General characteristics of participants across tertiles of nutrient-based pattern scores are indicated in Supplementary Table S1. Overall, regardless of any nutrient pattern, participants who adhered in higher MN intake were more likely to be younger, males, and non-Hispanic Whites, and were more likely to be current smokers and have a history of alcohol consumption. In addition, participants with adherence to higher VitAD-Ca-VB12 patterns were more educated.

**Table 2:** Factor loading matrix for the major micronutrient intake patterns in NHANES.

	VitB-mineral	VitAD-Ca-VB12	Antioxidant Vit
Vitamin E	0.534	0.133	0.591
Vitamin A	0.247	0.626	0.527
Vitamin B3	0.91	0.104	0.131
Vitamin B6	0.769	0.19	0.286
Vitamin B9	0.642	0.279	0.437
Vitamin B12	0.593	0.608	−0.019
Vitamin C	0.06	0.135	0.761
Vitamin D	0.178	0.855	0.039
Vitamin K	0.247	0.032	0.808
Ca	0.41	0.702	0.261
P	0.746	0.477	0.244
Fe	0.734	0.32	0.317
Zn	0.768	0.39	0.174
Se	0.798	0.286	0.138
Percent of variance explained	36.54%	19.37%	17.34%
Total variance explained	73.26%		

Factor loadings with absolute values ≥0.4 are highlighted with bold text and are considered significant contributors in each pattern.

### 3.3 Associations between single MN and sarcopenia

**Figure 3:**
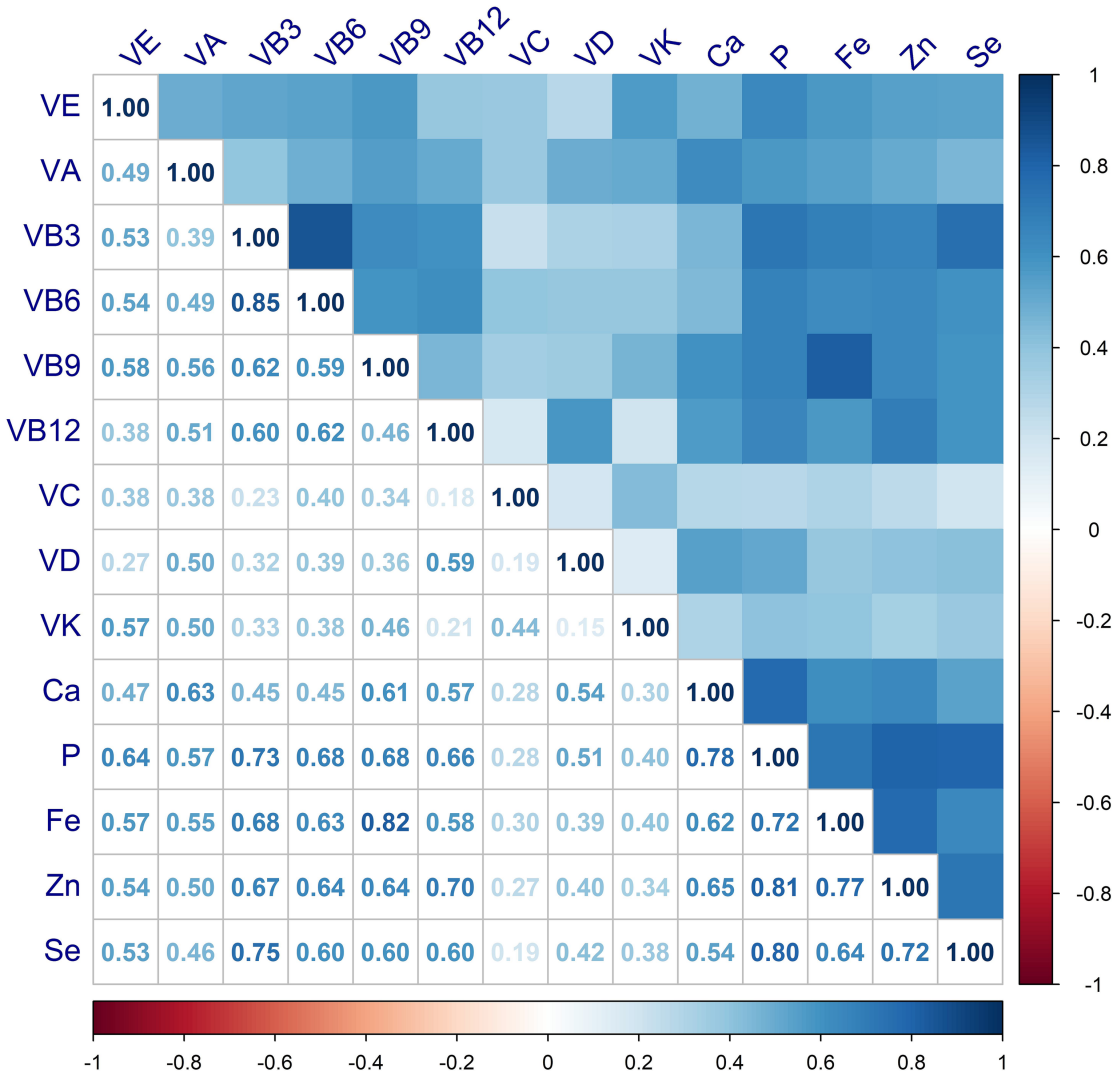
Correlations among intakes of micronutrients.

**Figure 4:**
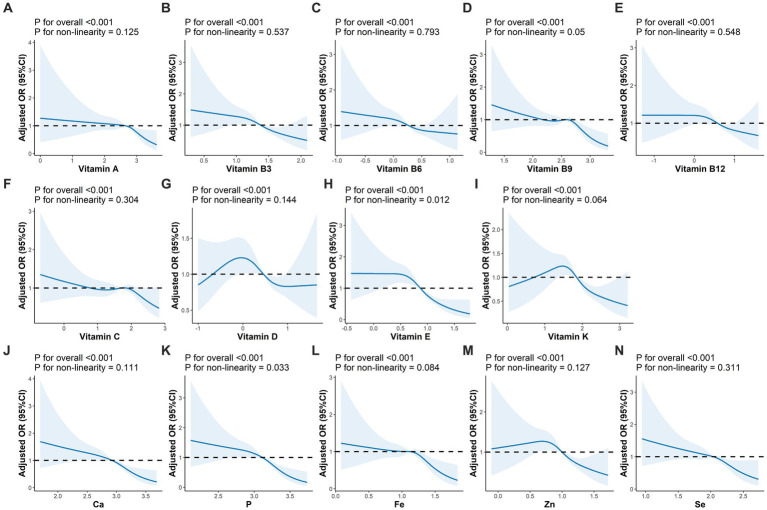
Adjusted OR (95%CI) between intakes of micronutrients and sarcopenia. **(A–N)** Represented the association of vitamin A, vitamin B3, vitamin B6, vitamin B9, vitamin B12, vitamin C, vitamin D, vitamin E, vitamin K, Ca, P, Fe, Zn, Se and sarcopenia, respectively. Adjusted for sex, age, race, BMI, education level, physical activity, smoking status, drinking history, hypertension, diabetes, cardiovasular disease, and cancer. BMI, body mass index.

The correlations between MN intake were shown in Figure 3, where VB shows moderate or strong correlation with minerals. For either nutrient intake pattern, subjects in the highest tertile had higher intakes of various vitamins and minerals compared to those in the lowest (Supplementary Table S2). We also found that those who adhered to a higher nutrient intake pattern were more likely to take in more macronutrients, such as energy and protein (Supplementary Table S3). The association between single MN and sarcopenia was illustrated in Supplementary Table S3, where, after adjusting for variables at baseline (Model 3), those with higher MN intake were less likely to have sarcopenia, except for VC and VD. After classification by tertiles, higher intake of MN other than VB6 and VC was associated with a lower prevalence of sarcopenia, as shown in Supplementary Figure S1. Furthermore, we observed a linear relationship between nutrients and sarcopenia, with the exception of VE and P, as shown in Figure 4.

### 3.4 Associations between nutrient patterns and sarcopenia

**Figure 5:**
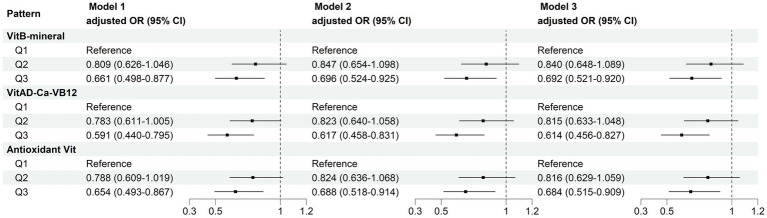
Multivariate logistic regression analyses of association between micronutrient patterns and sarcopenia. Model 1: adjusted for sex, age, race, and BMI. Model 2: adjusted for model 1 and education level, physical activity, smoking status, and drinking history. Model 3: adjusted for model 2 and hypertension, diabetes, cardiovasular disease, and cancer.

MN intake patterns with sarcopenia in the multivariate logistic regression analysis and with BMI-corrected ASM in the linear regression analysis were presented in Table 3. Results showed that after adjustment for confounders, participants adhering to higher VitB-mineral patterns, VitAD-Ca-VB12 patterns, and Antioxidant Vit patterns were associated with a reduced incidence of sarcopenia and a higher level of ASM compared to the lowest tertile (Figure 5; Table 3). Subgroup analyses showed that MN intake patterns in middle-aged females were significantly correlated with sarcopenia, which was at a lower risk in subjects with higher adherence (VitB-mineral, *p* = 0.008; VitAD-Ca-VB12, p = 0.008; Antioxidant Vit, *p* = 0.006), whereas no such association was observed in the other subgroups (Table 4).

**Table 3:** Logistic and linear regression analyses of association between micronutrient patterns and sarcopenia or BMI-corrected ASM.

	Model 1		Model 2		Model 3	
Sarcopenia	β/OR (95%CI)	*p*	β/OR (95%CI)	*p*	β/OR (95%CI)	*p*
*VitB-mineral*						
Q1	Reference	0.016	Reference	0.044	Reference	0.04
Q2	0.809 (0.626–1.046)	0.106	0.847 (0.654–1.098)	0.21	0.840 (0.648–1.089)	0.187
Q3	0.661 (0.498–0.877)	0.004	0.696 (0.524–0.925)	0.013	0.692 (0.521–0.920)	0.011
*VitAD-Ca-VB12*						
Q1	Reference	0.002	Reference	0.006	Reference	0.006
Q2	0.783 (0.611–1.005)	0.055	0.823 (0.640–1.058)	0.129	0.815 (0.633–1.048)	0.11
Q3	0.591 (0.440–0.795)	0.001	0.617 (0.458–0.831)	0.001	0.614 (0.456–0.827)	0.001
*Antioxidant Vit*						
Q1	Reference	0.011	Reference	0.034	Reference	0.031
Q2	0.788 (0.609–1.019)	0.07	0.824 (0.636–1.068)	0.144	0.816 (0.629–1.059)	0.126
Q3	0.654 (0.493–0.867)	0.003	0.688 (0.518–0.914)	0.01	0.684 (0.515–0.909)	0.009
**BMI-corrected ASM**						
*VitB-mineral*						
Q1	Reference		Reference		Reference	
Q2	0.012 (0.005–0.019)	0.001	0.010 (0.003–0.017)	0.006	0.010 (0.003–0.018)	0.004
Q3	0.030 (0.023–0.038)	<0.001	0.028 (0.020–0.035)	<0.001	0.028 (0.020–0.035)	<0.001
*VitAD-Ca-VB12*						
Q1	Reference		Reference		Reference	
Q2	0.012 (0.005–0.019)	0.001	0.010 (0.003–0.017)	0.006	0.010 (0.003–0.017)	0.005
Q3	0.030 (0.025–0.041)	<0.001	0.031 (0.023–0.039)	<0.001	0.031 (0.023–0.039)	<0.001
*Antioxidant Vit*						
Q1	Reference		Reference		Reference	
Q2	0.012 (0.005–0.019)	0.001	0.010 (0.003–0.017)	0.006	0.010 (0.003–0.018)	0.004
Q3	0.030 (0.022–0.038)	<0.001	0.028 (0.020–0.035)	<0.001	0.028 (0.020–0.035)	<0.001

Model 1: adjusted for sex, age, race, and BMI.Model 2: adjusted for model 1 and education level, physical activity, smoking status, and drinking history.Model 3: adjusted for model 2 and hypertension, diabetes, cardiovasular disease, and cancer.BMI, body mass index; ASM, appendicular skeletal muscle mass.

**Table 4:** Subgroup analyses of the association between micronutrient patterns and sarcopenia.

	Young male			Young female		
	Model 1	Model 2	Model 3	Model 1	Model 2	Model 3
*VitB-mineral*						
Q1	Reference	Reference	Reference	Reference	Reference	Reference
Q2	0.875 (0.475–1.611)	0.873 (0.471–1.618)	0.867 (0.467–1.611)	0.722 (0.398–1.309)	0.745 (0.403–1.376)	0.732 (0.393–1.362)
Q3	0.705 (0.399–1.245)	0.695 (0.391–1.236)	0.696 (0.39–1.242)	0.896 (0.454–1.767)	0.916 (0.457–1.837)	0.892 (0.440–1.807)
*p* for trend	0.453	0.43	0.441	0.562	0.641	0.616
*VitAD-Ca-VB12*						
Q1	Reference	Reference	Reference	Reference	Reference	Reference
Q2	0.914 (0.512–1.634)	0.925 (0.514–1.665)	0.918 (0.508–1.656)	0.633 (0.351–1.142)	0.648 (0.353–1.190)	0.628 (0.339–1.163)
Q3	0.618 (0.348–1.097)	0.609 (0.341–1.089)	0.613 (0.342–1.099)	0.912 (0.446–1.866)	0.922 (0.442–1.923)	0.919 (0.435–1.941)
*p* for trend	0.188	0.167	0.184	0.309	0.368	0.328
*Antioxidant Vit*						
Q1	Reference	Reference	Reference	Reference	Reference	Reference
Q2	0.888 (0.482–1.636)	0.891 (0.481–1.651)	0.888 (0.478–1.649)	0.675 (0.37–1.232)	0.697 (0.375–1.297)	0.682 (0.364–1.279)
Q3	0.713 (0.404–1.259)	0.706 (0.397–1.255)	0.708 (0.397–1.263)	0.878 (0.446–1.727)	0.898 (0.449–1.796)	0.872 (0.432–1.763)
*p* for trend	0.468	0.449	0.463	0.441	0.522	0.491

	Middle-aged male			Middle-aged female		
*VitB-mineral*						
Q1	Reference	Reference	Reference	Reference	Reference	Reference
Q2	1.04 (0.580–1.862)	1.064 (0.589–1.919)	1.116 (0.614–2.032)	0.821 (0.547–1.231)	0.855 (0.568–1.288)	0.857 (0.567–1.294)
Q3	0.972 (0.551–1.716)	1.038 (0.585–1.841)	1.087 (0.608–1.943)	0.310 (0.154–0.623)	0.322 (0.160–0.648)	0.328 (0.163–0.661)
*p* for trend	0.962	0.979	0.936	0.004	0.006	0.008
*VitAD-Ca-VB12*						
Q1	Reference	Reference	Reference	Reference	Reference	Reference
Q2	1.110 (0.634–1.944)	1.150 (0.653–2.026)	1.197 (0.674–2.124)	0.736 (0.492–1.101)	0.764 (0.509–1.147)	0.762 (0.506–1.147)
Q3	0.856 (0.481–1.523)	0.912 (0.510–1.630)	0.953 (0.529–1.716)	0.277 (0.125–0.612)	0.287 (0.130–0.635)	0.293 (0.133–0.649)
*p* for trend	0.557	0.631	0.627	0.005	0.007	0.008
*Antioxidant Vit*						
Q1	Reference	Reference	Reference	Reference	Reference	Reference
Q2	1.054 (0.588–1.886)	1.074 (0.595–1.936)	1.126 (0.619–2.047)	0.781 (0.519–1.176)	0.812 (0.538–1.226)	0.811 (0.536–1.229)
Q3	0.981 (0.556–1.73)	1.044 (0.589–1.851)	1.093 (0.612–1.952)	0.306 (0.152–0.615)	0.317 (0.157–0.637)	0.322 (0.16–0.649)
*p* for trend	0.955	0.973	0.926	0.004	0.005	0.006

Model 1: adjusted for sex, age, race, and BMI.Model 2: adjusted for model 1 and education level, physical activity, smoking status, and drinking history.Model 3: adjusted for model 2 and hypertension, diabetes, cardiovasular disease, and cancer.BMI, body mass index; ASM, appendicular skeletal muscle mass.

## 4 Discussion

In this cross-sectional study based on the US populations, we identified three major dietary MN patterns and examined their associations with sarcopenia risk and low muscle mass among US adults aged 20–59 years. Since food contains a variety of MNs and assessing the effect of one single MN on sarcopenia is incomplete, we considered overall nutrient intake patterns by PCA, and found that not only was most single MN intake associated with sarcopenia, but intakes in the higher tertiles of the VitB-mineral pattern, the VitAD-Ca-VB12 pattern, and the Antioxidant Vit pattern also showed significant correlations with reduced risk of sarcopenia and increased skeletal muscle mass. Our research aimed to explore the association of nutritional supplementation with reducing muscle atrophy and improving muscle functions.

The VitB-mineral pattern contained B vitamins, Fe, Zn and Se, and we found that adherence to the VitB-mineral pattern was associated with lower incidence of sarcopenia and higher muscle mass. As essential cofactors, B vitamins facilitate mitochondrial function and energy production needed for muscle protein synthesis (8, 29). They also aid neuromuscular signaling critical for muscle performance (30). Studies suggest B vitamins deficiency may lead to impaired mitochondrial function, reduced muscle anabolism and compromised neuromuscular integrity (8, 9). Most present studies have focused on the correlation between vitamins B6, B12, and folic acid and sarcopenia, with little work on the remaining B vitamins and sarcopenia. Verlaan et al. found that patients with sarcopenia had a 22% lower intake of VB12 and a 15% reduction in serum concentrations of VB12 (31). And higher intake of VB6 was also associated with a lower incidence of sarcopenia (32). In addition, higher levels of homocysteine (hcy) are associated with decreased muscle strength and gait speed, while VB6, VB9, and VB12 may be beneficial in the prevention of sarcopenia by promoting hcy metabolism and reducing plasma hcy levels (33). Zn and Se boost endogenous antioxidant enzymes to mitigate oxidative damage in muscle (7). Studies have shown that adequate intake of minerals may prevent muscle loss (34). Actually, we also included vitamin B1 (VB1) as an indicator during the initial selection, but results of the KMO test and variance interpretation were lower with the addition of VB1 in PCA, and it was unable to differentiate nutritional patterns for various MNs, which was not conducive to the subsequent analyses. Based on the above considerations, we excluded VB1. VB1 is a coenzyme of pyruvate dehydrogenase, which is associated with mitochondrial glucose metabolism and neurological functional integrity (8). Few studies have been conducted on the association between VB1 and sarcopenia, and the correlation between the two is still controversial (35, 36). In the present study, VB1 reduced the interpretation of the results, which we consider may be related to the inadequacy of the included population and recall bias during the investigation, thereby more large-scale studies are needed to explore the relationship between VB1 intake and muscle function. Overall, we found that increased intake of VitB-mineral patterns was associated with a decreased prevalence of sarcopenia, which suggested that B vitamins exerted a muscular protective effect, and that a proper increase in B vitamins intake could help to minimize muscle loss.

The VitAD-Ca-VB12 pattern represents bone and muscle-related nutrients. Although no correlation was observed between VD intake and sarcopenia in our study, adherence to higher VitAD-Ca-VB12 pattern was associated with a reduced risk of sarcopenia. VD helps maintain Ca homeostasis and regulates gene expression involved in muscle growth via VD receptors (VDR) (37). Ca acts by stimulating muscle contraction through its role in actin-myosin cycling, and activating mTOR pathway to stimulate protein synthesis (38). VD may enhance Ca absorption and bioavailability for optimal muscle effects (39). VD is a key modulator of the musculoskeletal system, and a study including 4,236 middle-aged and elderly Chinese residents showed that low VD levels were associated with age-related decreases in muscle mass, suggesting that VD supplementation may have a role in improving muscle function (40). Moreover, the impact of VD on sarcopenia depends on physical activity in older adults, involving a combined effect of VD and physical activity on the ubiquitination and degradation of muscle proteins (41). However, the association between VD and sarcopenia remains controversial, and our results suggested that increased food intakes rich in vitamin D and Ca may be effective in the prevention and treatment of sarcopenia.

The antioxidant vitamins pattern contained vitamins with redox-regulating capacities. We observed a negative correlation between antioxidant vitamins and sarcopenia, suggesting that oxidative stress plays a major role in the pathogenesis of sarcopenia. By suppressing chronic inflammation and muscle proteolysis, antioxidants help maintain redox homeostasis and mitigate oxidative damage associated with sarcopenia (42, 43). Specific antioxidants like VC and VE may protect against muscle protein oxidation and degradation, maintain muscle functions to the maximum extent, and promote glutathione antioxidant activity (15, 44).

Macronutrients are essential for organ and muscle functions, and several studies have shown that intakes of energy, protein, carbohydrates, and total fat are associated with sarcopenia (45, 46). Normally, these macronutrients stimulate muscle anabolism and decrease muscle catabolism, with protein being the most important nutrient for reducing muscle mass loss, which provides the essential amino acids required for the synthesis of muscle proteins. While insufficient intake of macronutrients leads to a supply–demand imbalance, with adverse effects on muscle health. We investigated differences in macronutrient intake across nutritional patterns, and the results showed that those adhering to all three nutritional patterns had significantly higher macronutrient intake, suggesting that macronutrients and MNs may interact together to have a cumulative effect on muscle mass and strength. A meta-analysis showed that combined supplementation with whey protein and VD increased lean body mass in an older population with sarcopenia or frailty, but had no significant effect in healthy older adults, in addition, taking nutritional supplements high in protein, energy, and VD improved muscle strength in patients with sarcopenia (47, 48). These studies support the combined roles of macronutrients and MNs in improving muscle anabolism and enhancing muscle functions, and additional clinical trials are warranted in the future to examine the effects of such nutritional patterns on sarcopenia. However, in our study, there was no significance of the effect of nutrient patterns on sarcopenia after adjusting for macronutrients, which was a pity. We speculate that this may be explained that a certain degree of subjectivity exists in the intake of nutrients, and that macronutrients like energy and carbohydrates are abundant and various in different foods, which may lead to a bias in the calculation of the combined intake of these nutrients. Another possible reason is the relatively inadequate sample size of the study population. Therefore, it needs to be validated by more studies after adjusting for macronutrients. In addition to this, there is no denying the important role of healthy dietary patterns in preserving muscle mass and maintaining muscle function, for example, Papadopoulou et al. conducted a systematic review to synthesize 10 studies on the Mediterranean diet and sarcopenia in a healthy elderly population for the first time, which showed that a Mediterranean diet pattern rich in monounsaturated fats, fibrous antioxidants, n-3 fatty acids, and anti-inflammatory micronutrients had a beneficial effect on physical function and muscle mass (49). Perry et al. demonstrated that the calorie-restricted Dietary Approaches to Stop Hypertension (DASH) dietary pattern may have improved muscle and cardiac metabolism by decreasing myostatin in older adults (50). The correlation between different dietary patterns and sarcopenia needs to be further investigated.

The associations of the three patterns with reduced sarcopenia likelihood suggest potentially synergistic effects between pattern components in protecting muscle health. B vitamins may interact with minerals to optimize mitochondrial and neurological functions to maintain muscle health (30, 51). Gkekas et al. demonstrated that supplementation with VD plus protein improves muscle strength in patients with sarcopenia, but it is not clear whether VD alone is beneficial (52). The active VD metabolite, 1,25-dihydroxyvitamin D (1,25-(OH)2D), binds to the VDR in the intestine to facilitate active Ca transport, and VD deficiency reduces intestinal absorption of Ca and P, which in turn leads to hypocalcemia and hypophosphatemia. Ca2+ in-flux is the initiator of muscle contraction, and VD deficiency is exacerbated by low Ca intake. VD may enhance beneficial effects of Ca on muscle by improving its bioavailability and potentiating its anabolic actions (53). VA, as fat-soluble vitamins, can promote VD to exert their biological activities, and studies have shown that VDR needs to bind to the retinoid X receptor (RXR) to form a heterodimer to activate the transcription of target genes. The two share combined roles in anti-infective properties, promotion of bone development, amelioration of iron-deficiency anemia, and inhibition of cancer (54). VB12 may play a role in lowering inflammation levels in conjunction with VD (55). Therefore, preventing muscle atrophy and counteracting sarcopenia can only be achieved if all of these nutrients are adequate (53), demonstrating the necessity of increasing dietary intake of VA,VD, Ca and VB12. Combinations of antioxidant vitamins may collectively optimize redox regulation and attenuate muscle catabolism (56), while individual MNs do not exhibit such correlations (57, 58). These findings were similar to our study, that no association was observed between VC and sarcopenia. However, our results suggest a linear dose–response relationship between the intake of most MNs and the prevalence of sarcopenia, providing novel evidence that overall adequate intake of these nutrients and their combinations through dietary balance could be used as part of a strategy to reduce age-related muscle deterioration.

Most previous studies have targeted older individuals, as they are characterized by a predisposition to malnutrition, susceptibility to oxidative defenses, and decreased neurological integrity. Our study involved adults aged 20–59 years and identified a significant association between MN intake and sarcopenia in middle-aged females, not in males or younger adults. The accelerated muscle decline in perimenopausal women could be attributed to estrogen reduction, heightened oxidative stress and inflammation (59, 60). A longitudinal study showed that the transition from perimenopause to early postmenopause was associated with loss of muscle mass at multiple anatomical levels, which was independent of aging (61). However, age and sex differences in lifestyle factors like physical activity may also contribute to the divergent findings (62). Furthermore, constitutional variations in antioxidant capacity, vitamin D status, and calcium homeostasis may underlie the differential effects (63). Younger adults likely have better intrinsic nutrient status and resilience compared to midlife women (64). Optimizing MN intake could help compensate for age-related changes and counteract sarcopenic progression in midlife women (65). These results provide implications for tailoring MN intake recommendations by specific life stages and conditions.

The findings underscore the importance of overall adequate intake of B vitamins, minerals, VD, Ca, and antioxidant vitamins in mitigating age-related muscle deterioration. Translating these results, tailored optimization of beneficial MN combinations through dietary balance and supplementation should be considered in formulating stage-specific intake guidance and food policies, to promote public muscle health across adulthood. Several limitations of our study need to be recognized. Firstly, this was a cross-sectional study with no strong causality; secondly, MN intake was obtained by the first 24-h recall interview, which may have recall bias; in addition, macronutrients were not adjusted as confounders, and VB1 was not incorporated in PCA, both of which should be included in the subsequent study, and finally, data extraction was based on surveys at baseline, which did not take into account the effects of nutrient patterns on sarcopenia at multiple time points.

In conclusion, this study identified three dietary MN patterns associated with reduced sarcopenia and greater muscle mass in US adults, with significant associations in middle-aged females. This suggests that MNs interact with each other along with their respective functions, and provides a novel perspective to explore the combined intake effects of multiple MNs on reducing muscle wasting and maintaining physical functions. Ensuring sufficient intake of these beneficial MNs may aid sarcopenia prevention across life stages. Future prospective studies are still warranted to confirm this and to elucidate the potential mechanisms of MN interactions.

## Data availability statement

The original contributions presented in the study are included in the article/Supplementary material, further inquiries can be directed to the corresponding authors.

## Ethics statement

The studies involving humans were approved by the National Center for Health Statistics (NCHS) Research Ethics Review Board. The studies were conducted in accordance with the local legislation and institutional requirements. The participants provided their written informed consent to participate in this study.

## Author contributions

YL: Conceptualization, Methodology, Validation, Writing – original draft. XL: Conceptualization, Methodology, Writing – original draft. LD: Software, Visualization, Writing – original draft. YZ: Methodology, Writing – review & editing. YH: Formal analysis, Writing – review & editing. WL: Supervision, Writing – review & editing. JC: Supervision, Writing – review & editing.

## Footnotes

^1^ http://www.cdc.gov/nchs/nhanes

^2^ https://search.cdc.gov/search/index.html?query=DXA&siteLimit=NCHS&dpage=1
