# Supplementary material for: Associations of micronutrient dietary patterns with sarcopenia among US adults: a population-based study
Source: Front Nutr. 2024 Feb 12;11:1301831. doi: 10.3389/fnut.2024.1301831 (PMC10894935; doi:10.3389/fnut.2024.1301831)
Supplement: Supplementary file 1 [file Data_Sheet_1.docx]

Supplementary Material

Associations of micronutrient dietary with sarcopenia among US adults: a population-based study

Yining Liu^1†^, Xiangliang Liu^1†^, Linnan Duan^2^, Yixin Zhao^1^, Yuwei He^1^, Wei Li^1*^, Jiuwei Cui^1*^

*** Correspondence:** Wei Li ,[liwei66@jlu.edu.cn](mailto:liwei66@jlu.edu.cn); JiuweiCui, [cuijw@jlu.edu.cn](mailto:cuijw@jlu.edu.cn)

## Supplementary Figures


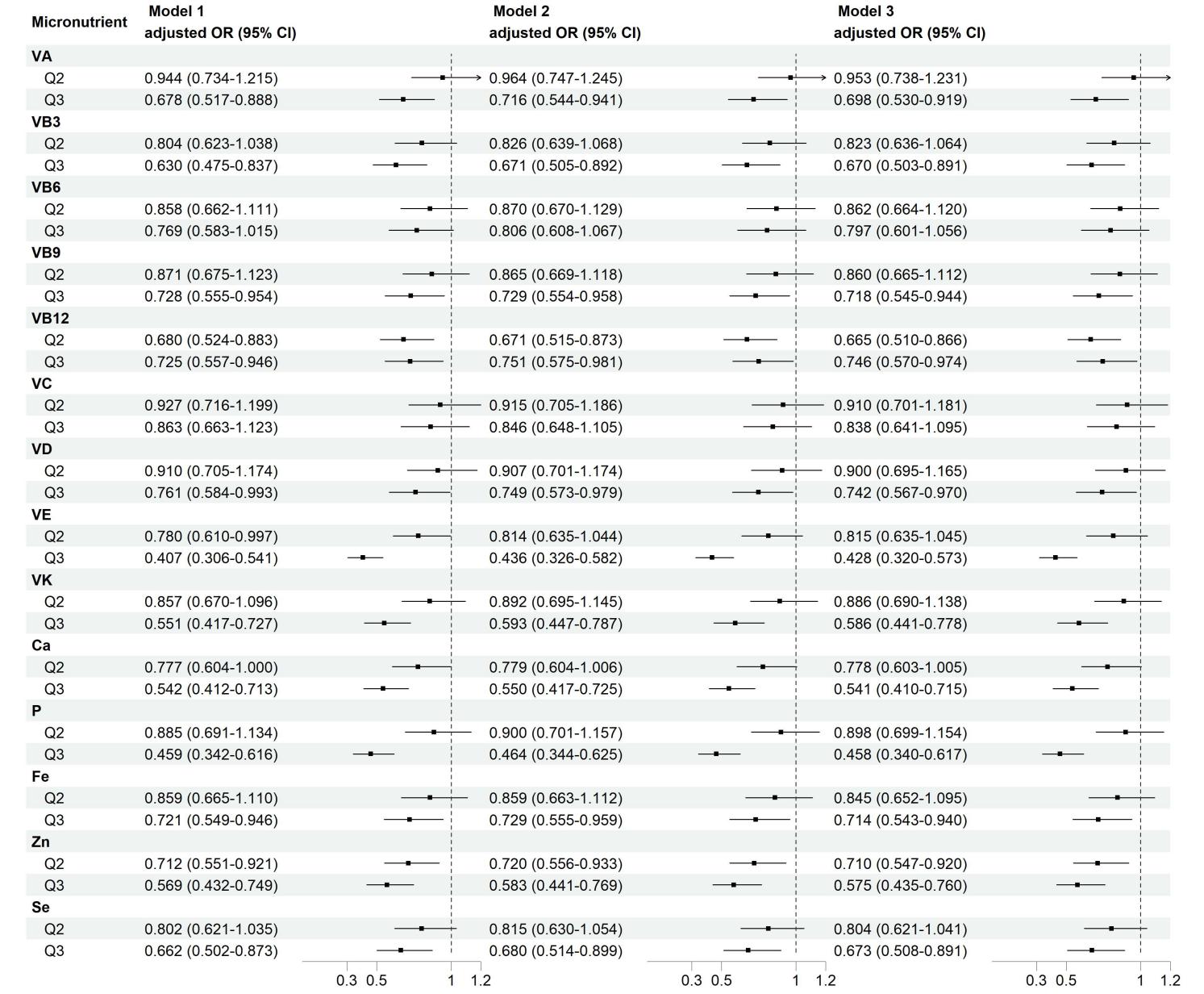
**Supplementary Figure 1.** Multivariate logistic regression analyses of association between micronutrient intakes and sarcopenia. Q1 as reference. Model 1: adjusted for sex, age, race, and BMI. Model 2: adjusted for model 1 and education level, physical activity, smoking status, and drinking history. Model 3: adjusted for model 2 and hypertension, diabetes, cardiovasular disease, and cancer.

## Supplementary Figures

**Supplementary Table 1.** Baseline characteristics of the study population across tertiles of micronutrient patterns

|  | VitB-mineral | | | | VitAD-Ca-VB12 | | | | Antioxidant Vit | | | |
| --- | --- | --- | --- | --- | --- | --- | --- | --- | --- | --- | --- | --- |
| **Characteristics** | Q1 | Q2 | Q3 | P | Q1 | Q2 | Q3 | P | Q1 | Q2 | Q3 | P |
| **Median (IQR)** |  |  |  |  |  |  |  |  |  |  |  |  |
| Age (y) | 40.0 (21.0) | 40.0 (19.0) | 36.0 (19.0) | <0.001 | 40.0 (21.0) | 40.0 (19.0) | 36.0 (19.0) | <0.001 | 40.0 (21.0) | 40.0 (20.0) | 36.0 (19.0) | <0.001 |
| BMI, kg/m2 | 27.5 (9.6) | 27.7 (8.0) | 27.4 (7.8) | 0.148 | 27.7 (9.7) | 27.6 (7.9) | 27.3 (7.8) | 0.156 | 27.6 (9.7) | 27.6 (8.0) | 27.4 (7.8) | 0.159 |
| **N (%)** |  |  |  |  |  |  |  |  |  |  |  |  |
| Sex, Male | 532 (30.4%) | 873 (49.7%) | 1293 (73.9%) | <0.001 | 554 (30.8%) | 963 (50.4%) | 1181 (76.4%) | <0.001 | 536 (30.3%) | 870 (50.0%) | 2698 (51.3%) | <0.001 |
| Race |  |  |  | 0.02 |  |  |  | 0.011 |  |  |  | 0.019 |
| Mexican American | 215 (12.3%) | 199 (11.3%) | 251 (14.3%) |  | 220 (12.2%) | 220 (11.5%) | 225 (14.6%) |  | 216 (12.2%) | 198 (11.4%) | 251 (14.4%) |  |
| Non-Hispanic White | 664 (37.9%) | 714 (40.7%) | 719 (41.1%) |  | 686 (38.1%) | 777 (40.7%) | 634 (41.0%) |  | 671 (38.0%) | 709 (40.7%) | 717 (41.0%) |  |
| Non-Hispanic Black | 401 (22.9%) | 381 (21.7%) | 369 (21.1%) |  | 411 (22.8%) | 406 (21.3%) | 334 (21.6%) |  | 406 (23.0%) | 376 (21.6%) | 369 (21.1%) |  |
| Others | 470 (26.9%) | 462 (26.3%) | 411 (23.5%) |  | 484 (26.9%) | 507 (26.5%) | 352 (22.8%) |  | 475 (26.9%) | 458 (26.3%) | 410 (23.5%) |  |
| Education level |  |  |  | 0.106 |  |  |  | 0.034 |  |  |  | 0.113 |
| Below high school | 323 (18.5%) | 288 (16.4%) | 300 (17.1%) |  | 333 (18.5%) | 304 (15.9%) | 274 (17.3%) |  | 325 (18.4%) | 286 (16.4%) | 300 (17.2%) |  |
| High school | 370 (21.1%) | 357 (20.3%) | 405 (23.1%) |  | 381 (21.2%) | 391 (20.5%) | 360 (23.3%) |  | 375 (21.2%) | 353 (20.3%) | 404 (23.1%) |  |
| Above high school | 1057 (60.4%) | 1111 (63.3%) | 1045 (59.7%) |  | 1087 (60.4%) | 1215 (63.6%) | 911 (59.0%) |  | 1068 (60.4%) | 1102 (63.3%) | 1043 (59.7%) |  |
| Smoking |  |  |  | <0.001 |  |  |  | <0.001 |  |  |  | <0.001 |
| Never | 1113 (63.6%) | 1044 (59.5%) | 974 (55.7%) |  | 1144 (63.5%) | 1132 (59.3%) | 855 (55.3%) |  | 1124 (63.6%) | 1036 (59.5%) | 971 (55.6%) |  |
| Ever | 258 (14.7%) | 291 (16.6%) | 327 (18.7%) |  | 264 (14.7%) | 324 (17.0%) | 288 (18.6%) |  | 260 (14.7%) | 289 (16.6%) | 327 (18.7%) |  |
| Current | 379 (21.7%) | 421 (24.0%) | 449 (25.7%) |  | 393 (21.8%) | 454 (23.8%) | 402 (26.0%) |  | 384 (21.7%) | 416 (23.9%) | 449 (25.7%) |  |
| Drinking (yes) | 1229 (70.2%) | 1369 (78.0%) | 1450 (82.9%) | <0.001 | 1265 (70.2%) | 1493 (78.2%) | 1290 (83.5%) | <0.001 | 1241 (70.2%) | 1359 (78.1%) | 1448 (82.9%) | <0.001 |
| Recreational physical activity | |  |  | 0.151 |  |  |  | 0.563 |  |  |  | 0.135 |
| Sedentary | 954 (54.5%) | 981 (55.9%) | 953 (54.5%) |  | 988 (54.9%) | 1048 (54.9%) | 852 (55.1%) |  | 965 (54.6%) | 972 (55.8%) | 951 (54.4%) |  |
| Insufficient | 385 (22.0%) | 417 (23.7%) | 373 (21.3%) |  | 393 (21.8%) | 452 (23.7%) | 330 (21.4%) |  | 387 (21.9%) | 415 (23.8%) | 373 (21.4%) |  |
| Moderate | 227 (13.0%) | 193 (11.0%) | 232 (13.3%) |  | 234 (13.0%) | 219 (11.5%) | 199 (12.9%) |  | 230 (13.0%) | 191 (11.0%) | 231 (13.2%) |  |
| High | 184 (10.5%) | 165 (9.4%) | 192 (11.0%) |  | 186 (10.3%) | 191 (10.0%) | 164 (10.6%) |  | 186 (10.5%) | 163 (9.4%) | 192 (11.0%) |  |
| Hypertension (yes) | 462 (26.4%) | 430 (24.5%) | 420 (24.0%) | 0.222 | 473 (26.3%) | 470 (24.6%) | 369 (23.9%) | 0.257 | 466 (26.4%) | 426 (24.5%) | 420 (24.0%) | 0.24 |
| Diabetes (yes) | 224 (12.8%) | 242 (13.8%) | 208 (11.9%) | 0.244 | 230 (12.8%) | 266 (13.9%) | 178 (11.5%) | 0.109 | 226 (12.8%) | 240 (13.8%) | 208 (11.9%) | 0.252 |
| Cardiovasular disease (yes) | 37 (2.1%) | 26 (1.5%) | 33 (1.9%) | 0.365 | 37 (2.1%) | 30 (1.6%) | 29 (1.9%) | 0.538 | 37 (2.1%) | 26 (1.5%) | 33 (1.9%) | 0.404 |
| Cancer (yes) | 75 (4.3%) | 69 (3.9%) | 59 (3.4%) | 0.368 | 76 (4.2%) | 75 (3.9%) | 52 (3.4%) | 0.434 | 76 (4.3%) | 68 (3.9%) | 59 (3.4%) | 0.364 |

Values were presented as Median (IQR) or Number (%). BMI, body mass index. IQR, interquartile range.

**Supplementary Table 2. Differences of micronutrient intakes across tertiles of micronutrient patterns**

|  | VitB-mineral | | | | VitAD-Ca-VB12 | | | | Antioxidant Vit | | | |
| --- | --- | --- | --- | --- | --- | --- | --- | --- | --- | --- | --- | --- |
| **Mean±SE** | Q1 | Q2 | Q3 | P | Q1 | Q2 | Q3 | P | Q1 | Q2 | Q3 | P |
| VA (mcg) | 2.500±0.011 | 2.632±0.009 | 2.690±0.010 | <0.001 | 2.502±0.011 | 2.642±0.009 | 2.688±0.011 | <0.001 | 2.500±0.011 | 2.633±0.009 | 2.690±0.010 | <0.001 |
| VB3 (mg) | 1.121±0.005 | 1.379±0.003 | 1.604±0.004 | <0.001 | 1.126±0.005 | 1.393±0.003 | 1.620±0.004 | <0.001 | 1.123±0.005 | 1.380±0.003 | 1.605±0.004 | <0.001 |
| VB6 (mg) | 0.028±0.006 | 0.263±0.004 | 0.480±0.005 | <0.001 | 0.031±0.006 | 0.276±0.004 | 0.496±0.006 | <0.001 | 0.029±0.006 | 0.264±0.004 | 0.480±0.005 | <0.001 |
| VB9 (mcg) | 2.345±0.007 | 2.549±0.005 | 2.721±0.005 | <0.001 | 2.349±0.007 | 2.561±0.005 | 2.732±0.006 | <0.001 | 2.347±0.007 | 2.550±0.005 | 2.721±0.005 | <0.001 |
| VB12 (mcg) | 0.292±0.010 | 0.555±0.008 | 0.791±0.008 | <0.001 | 0.297±0.009 | 0.570±0.007 | 0.806±0.009 | <0.001 | 0.293±0.009 | 0.555±0.008 | 0.792±0.008 | <0.001 |
| VC (mg) | 1.584±0.014 | 1.643±0.013 | 1.635±0.014 | 0.005 | 1.587±0.014 | 1.645±0.013 | 1.630±0.015 | 0.008 | 1.586±0.014 | 1.641±0.013 | 1.636±0.014 | 0.009 |
| VD (mcg) | 0.284±0.012 | 0.407±0.012 | 0.480±0.013 | <0.001 | 0.285±0.012 | 0.419±0.012 | 0.479±0.014 | <0.001 | 0.284±0.012 | 0.409±0.012 | 0.480±0.013 | <0.001 |
| VE (mg) | 0.678±0.007 | 0.871±0.006 | 1.021±0.006 | <0.001 | 0.682±0.007 | 0.880±0.006 | 1.031±0.007 | <0.001 | 0.681±0.007 | 0.870±0.006 | 1.020±0.006 | <0.001 |
| VK (mcg) | 1.743±0.012 | 1.879±0.010 | 1.950±0.009 | <0.001 | 1.746±0.012 | 1.885±0.009 | 1.952±0.009 | <0.001 | 1.745±0.012 | 1.879±0.010 | 1.949±0.009 | <0.001 |
| Ca (mg) | 2.778±0.007 | 2.910±0.006 | 3.018±0.006 | <0.001 | 2.779±0.007 | 2.921±0.006 | 3.023±0.007 | <0.001 | 2.778±0.007 | 2.912±0.006 | 3.018±0.006 | <0.001 |
| P (mg) | 2.922±0.005 | 3.117±0.003 | 3.271±0.004 | <0.001 | 2.925±0.005 | 3.128±0.003 | 3.281±0.004 | <0.001 | 2.923±0.005 | 3.118±0.003 | 3.271±0.004 | <0.001 |
| Fe (mg) | 0.911±0.005 | 1.125±0.004 | 1.301±0.005 | <0.001 | 0.915±0.005 | 1.137±0.004 | 1.311±0.005 | <0.001 | 0.912±0.005 | 1.126±0.004 | 1.301±0.005 | <0.001 |
| Zn (mg) | 0.772±0.005 | 1.000±0.004 | 1.196±0.005 | <0.001 | 0.777±0.005 | 1.011±0.004 | 1.211±0.005 | <0.001 | 0.774±0.005 | 1.001±0.004 | 1.196±0.005 | <0.001 |
| Se (mcg) | 1.803±0.006 | 2.037±0.004 | 2.222±0.004 | <0.001 | 1.807±0.006 | 2.051±0.003 | 2.234±0.005 | <0.001 | 1.804±0.006 | 2.039±0.004 | 2.222±0.004 | <0.001 |

Values were presented as Mean±SE. SE, Standard Error.

**Supplementary Table 3. Differences of micronutrient intakes across tertiles of micronutrient patterns**

|  | VitB-mineral | | | | VitAD-Ca-VB12 | | | | Antioxidant Vit | | | |
| --- | --- | --- | --- | --- | --- | --- | --- | --- | --- | --- | --- | --- |
| **Mean±SE** | Q1 | Q2 | Q3 | P | Q1 | Q2 | Q3 | P | Q1 | Q2 | Q3 | P |
| Energy (kcal) | 3.148±0.004 | 3.321±0.003 | 3.468±0.004 | <0.001 | 3.151±0.004 | 3.330±0.003 | 3.478±0.004 | <0.001 | 3.149±0.004 | 3.322±0.003 | 3.468±0.004 | <0.001 |
| Protein (gm) | 1.671±0.005 | 1.899±0.003 | 2.077±0.004 | <0.001 | 1.675±0.005 | 1.910±0.003 | 2.089±0.004 | <0.001 | 1.673±0.005 | 1.900±0.003 | 2.077±0.004 | <0.001 |
| Carbohydrate (gm) | 2.249±0.005 | 2.391±0.004 | 2.520±0.004 | <0.001 | 2.251±0.005 | 2.339±0.004 | 2.529±0.005 | <0.001 | 2.250±0.005 | 2.392±0.004 | 2.520±0.004 | <0.001 |
| Total fat (gm) | 1.679±0.006 | 1.879±0.005 | 2.024±0.005 | <0.001 | 1.683±0.006 | 1.889±0.005 | 2.033±0.005 | <0.001 | 1.681±0.006 | 1.880±0.005 | 2.024±0.005 | <0.001 |

Values were presented as Mean±SE. SE, Standard Error.

**Supplementary Table 4.** Multivariate adjusted OR (95% CI) of sarcopenia in relation to micronutrient patterns among participants

|  | Model 1 | | Model 2 | | Model 3 | |
| --- | --- | --- | --- | --- | --- | --- |
|  | OR (95%CI) | P | OR (95%CI) | P | OR (95%CI) | P |
| VA | 0.723 (0.573-0.912) | 0.006 | 0.749 (0.590-0.952) | 0.018 | 0.734 (0.577-0.932) | 0.011 |
| VB3 | 0.458 (0.297-0.708) | <0.001 | 0.491 (0.316-0.763) | 0.002 | 0.481 (0.309-0.749) | 0.001 |
| VB6 | 0.634 (0.432-0.933) | 0.021 | 0.665 (0.449-0.985) | 0.042 | 0.647 (0.436-0.961) | 0.031 |
| VB9 | 0.596 (0.411-0.863) | 0.006 | 0.610 (0.420-0.886) | 0.009 | 0.596 (0.410-0.867) | 0.007 |
| VB12 | 0.701 (0.583-0.913) | 0.008 | 0.730 (0.559-0.954) | 0.021 | 0.724 (0.554-0.946) | 0.018 |
| VC | 0.873 (0.730-1.044) | 0.137 | 0.865 (0.721-1.038) | 0.119 | 0.857 (0.714-1.029) | 0.098 |
| VD | 0.851 (0.696-1.040) | 0.115 | 0.840 (0.686-1.029) | 0.092 | 0.832 (0.679-1.020) | 0.077 |
| VE | 0.333 (0.238-0.465) | <0.001 | 0.359 (0.254-0.506) | <0.001 | 0.351 (0.249-0.496) | <0.001 |
| VK | 0.600 (0.468-0.770) | <0.001 | 0.634 (0.491-0.818) | <0.001 | 0.622 (0.481-0.804) | <0.001 |
| Ca | 0.412 (0.286-0.593) | <0.001 | 0.420 (0.290-0.608) | <0.001 | 0.414 (0.286-0.599) | <0.001 |
| P | 0.319 (0.203-0.502) | <0.001 | 0.333 (0.210-0.528) | <0.001 | 0.325 (0.205-0.517) | <0.001 |
| Fe | 0.547 (0.356-0.838) | 0.006 | 0.560 (0.364-0.861) | 0.008 | 0.540 (0.351-0.833) | 0.005 |
| Zn | 0.466 (0.306-0.708) | <0.001 | 0.443 (0.293-0.671) | <0.001 | 0.454 (0.298-0.691) | <0.001 |
| Se | 0.501 (0.330-0.759) | 0.001 | 0.507 (0.333-0.774) | 0.002 | 0.498 (0.326-0.761) | 0.001 |

Model 1: adjusted for sex, age, race, and BMI.

Model 2: adjusted for model 1 and education level, physical activity, smoking status, and drinking history.

Model 3: adjusted for model 2 and hypertension, diabetes, cardiovasular disease, and cancer
